# Diagnostic yield of nontuberculous mycobacteria in patients booked for endoscopy at the University Teaching Hospital, Lusaka

**DOI:** 10.1186/s13104-016-2329-3

**Published:** 2017-01-07

**Authors:** Gershom Chongwe, Charles Michelo, Paul Kelly

**Affiliations:** 1 Department of Public Health, School of Medicine, University of Zambia, Box 50110, Lusaka, Zambia; 2 Tropical Gastroenterology Unit, School of Medicine, Department of Internal Medicine, University of Zambia, Box 50110, Lusaka, Zambia; 3 Barts and The London School of Medicine and Dentistry, London, UK

**Keywords:** Nontuberculous mycobacteria, Gastrointestinal, Endoscopy, Colonoscopy

## Abstract

**Background:**

The intestinal carriage of nontuberculous mycobacteria (NTM) is associated with disease, especially in severely immunocompromised individuals. These organisms, although often considered contaminants, have been known to cause various types of illnesses. We aimed to determine the prevalence of and associated factors for NTM among patients booked for colonoscopy at the University Teaching Hospital (UTH) in Lusaka.

**Methods:**

We randomly recruited 97 patients attending routine endoscopy procedures between November 2012 and October 2013 and after consent, administered a structured questionnaire. We collected stool and intestinal lavage samples, as well as biopsy samples from the descending colon and the caecal area during the endoscopy procedure. Samples were cultured using the mycobacteria growth indicator tube (MGIT) method followed by the GenoType Mycobacterium CM/AS assay for identification of NTM. Results were expressed as means and standard deviations; proportions were expressed as percentages with corresponding 95% confidence intervals. We used Fisher’s exact Chi square test for cross-tabulations where appropriate. All statistical tests were two-sided, with a significance level set at p < 0.05.

**Results:**

Out of the 97 patients, 45 (46.4%) were female and 52 (53.6%) were males with mean ages 49.1 (±16.7, range 24–85) and 44.4 (±15.0, range 18–80) years respectively. The prevalence of NTM was 7.2% (95% CI 1.9–12.4), while that of *Mycobacterium tuberculosis* (MTB) was 6.2% (95% CI 2.3–13.0). Carriage of NTM was not significantly associated with age, sex or presenting symptoms such as diarrhoea, abdominal pain, weight loss as well as HIV status. There were no identifiable predictors of NTM carriage.

**Conclusion:**

The results have shown that NTM and MTB are present in the intestines of the patients booked for colonoscopy at the University Teaching Hospital in Lusaka, but their presence is not related to presenting symptoms. Given that this may be an indicator of a bigger burden of NTM in this population, there is a need to explore this burden and the contribution it could have on abdominal disease in general as well as examine potential factors that might be important predictors.

## Background

The intestinal carriage of nontuberculous mycobacteria (NTM) is associated with disease in both immunocompetent as well as immunocompromised individuals [1]. Nontuberculous mycobacteria (NTM) are a group of *Mycobacterium* species different from those belonging to the *Mycobacterium tuberculosis* complex (MTB) and *M. leprae*. There are currently over 150 NTM species recognised [2]. The organisms, although frequently dismissed as mere contaminants, have been known to cause various types of illnesses affecting the skin, lungs, intestines, the joints as well as the blood stream. There has been an increased incidence of NTM since the advent of HIV, partly due to HIV but also because of increased awareness [3–5].

Many NTM are free-living organisms which have been isolated in a wide range of environments, including water, soil, dust and aerosols. In general, disease due to NTM is more common in individuals with suppressed local or systemic immunity than those with normal immunity. However, some studies have found an increased incidence of NTM disease in the elderly [6]. Some of the conditions predisposing to systemic, respiratory and abdominal NTM disease include HIV, emphysema, pneumoconiosis, cystic fibrosis, previous gastrectomy, continuous ambulatory dialysis and chronic alcoholism [7–9]. In the gastrointestinal tract, both *M. tuberculosis* and NTM can cause abdominal pain, ascites, chronic diarrhoea and other clinical manifestations [10].

Gastrointestinal (GI) disease is characterised by invasion of Peyer’s patches and mesenteric lymph nodes of the small intestine, leading to foamy histiocytes and mycobacteria-laden macrophages within the lamina propria of the intestinal mucosa. Symptoms include diffuse abdominal pain, weight loss, fever and/or diarrhoea. Findings on endoscopy include multiple raised nodules or normal-appearing mucosa. Other findings may include ulceration, erythema, oedema, friability, reduced mucosal vascularity, stricture, and aphthous erosions [11, 12]. The prevalence of NTM in intestinal specimens such as stool, intestinal lavage and colon biopsies is unknown.

Little is known about the role of NTM in the aetiology of abdominal disease in Zambian patients. Further it has been suggested that the burden of nontuberculous mycobacteria in Zambia, some of which may present with abdominal symptoms, may be underestimated [13]. This article presents the results of a descriptive study of carriage rates of NTM as well as factors associated with their carriage in patients booked for endoscopy at the University Teaching Hospital (UTH) in Lusaka, Zambia.

## Methods

### Design and study population

We randomly sampled patients booked for endoscopy at the University Teaching Hospital in Lusaka between November 2012 and October 2013 in this cross-sectional survey. All patients who were 18 years and older who were booked for either colonoscopy or flexible sigmoidoscopy were eligible to take part in the study. Patients who were on treatment for TB, those who were found to have cancer lesions on endoscopy or those with debilitating conditions were not considered for recruitment.

### Sample collection and processing

Consenting patients were asked to answer questions on their demographic characteristics, symptoms and drug history using a structured questionnaire, which took up to 10 min. Blood was collected for HIV and haemoglobin tests. HIV testing was done using the recommended national testing algorithm in Zambia, which follows the standard two test algorithm that starts with the Allere Determine® HIV1/2 test kit (Abbott, Japan) followed by the Uni-Gold™ Recombigen HIV-1/2 (Trinity Biotech) if positive [14]. The two tests showed an agreement of 100%. Furthermore, patients who wanted to know their results were offered counselling services before the results were shared with them.

Samples of stool and intestinal lavage were collected from the patients before they underwent their scheduled procedure. After specimen collection, the patients underwent either a flexible sigmoidoscopy or a colonoscopy, during which descending colon biopsy and/or caecal biopsy samples were obtained from eligible patients. The biopsy samples were collected in 2 ml cryovials filled with saline. These samples were then used to test for the presence of mycobacteria as described below.

The BACTEC mycobacteria growth indicator tubes (MGIT) liquid culture method was used to establish the presence of nontuberculous mycobacteria from the biopsies and lavage fluids after decontamination with 6% NaOH. The biopsy samples were first homogenised before being transferred to 50 ml Falcon centrifuge tubes for processing, in much the same way as sputum samples are processed. For each batch of samples processed, care was taken to ensure that a positive (H37Rv laboratory stock) and a negative control (phosphate buffer, pH 6.8) were included. The samples were incubated in the MGIT 960 machine at 37 °C for up to 42 days. No additional supplements for isolation of fastidious mycobacteria were used. Samples from positive MGIT tubes were inoculated onto blood agar plates to check for contamination, followed by Ziehl Neelsen (ZN) staining and the Capilia assay (Capilia® TB, TAUNS Laboratories Inc, Shizuoka, Japan) to confirm *M. tuberculosis* [15, 16]. For identification, we used the GenoType mycobacterium CM/AS (Hain Lifescience, GmbH, Germany) assay according to manufacturer’s specifications.

### DNA extraction

To extract DNA, 1 ml was drawn from a positive MGIT tube into a container and centrifuged at 10,000*g* for 15 min in a class II safety cabinet. The supernatant was discarded and the pellet was re-suspended in 300 µl of molecular grade water. Following that, the suspension was sonicated for 15 min, followed by incubation in a water bath for 20 min at 95 °C as per manufacturer instructions [17].

In order to test for contamination of water sources with NTM, we collected water samples in 50 ml Falcon tubes at different time-points in the endoscopy unit (where the sigmoidoscopy and colonoscopies took place) as well as in the TB laboratory. These samples were centrifuged at 3000 rpm for 15 min, supernatant discarded and 5 ml of the remaining fluid was processed for mycobacteria using the MGIT method as described above.

### Statistical analysis

A structured questionnaire was designed to collect demographic characteristics, symptoms and drug history. The data was entered into a questionnaire created in Epidata statistical software on a panel that matched the unique identifier for the individual’s demographic data, then analysed using STATA (Version 11.2, StataCorp, College Station, Texas). Sample descriptions were expressed as means with their respective standard deviations; whereas proportions (e.g. prevalence) were expressed as percentages with corresponding 95% confidence intervals. We used Fisher’s exact Chi square test for cross-tabulations where appropriate. Tests for normality of age and other continuous variables were done using the Shapiro–Wilks test. Mantel–Haenszel tests were used to test for interaction and confounding. Multivariable logistic regression was used to look for predictors for carriage of mycobacteria. A p < 0.05 was considered statistically significant. The model comprised age, occupation, residence, level of education as well as presenting symptoms such as abdominal pain, diarrhoea, vomiting, fever and weight loss. The Akaike (AIC) and Bayesian (BIC) information criteria were used for model diagnostics. The outcome variable was carriage of any nontuberculous mycobacteria. Carriage of MTB was the secondary outcome variable.

### Ethical considerations

All the respondents provided written informed consent before participating in the study. Although unlinked HIV testing was done, patients who wanted to know their results were made to undergo counselling according to standard national guidelines before results were shared with them. The proposal was approved by the University of Zambia Biomedical Research Ethics Committee (Reference no. 015-07-12).

## Results

### Study characteristics

Out of 97 participants recruited, 45 (46.4%) were female and 52 (53.6%) were male, with mean age 46.6 years (±15.9). The age distribution by sex was similar in both females and males (mean 49.1 ± 16.7 vs 44.4 ± 14.9 years, p = 0.15) respectively. The refusal rate was 4%.

### Medical characteristics

Abdominal pain (60.8%, 95% CI 50.4–70.6) was the most common indication for colonoscopy, followed by passing blood in stool (39.2%, 95% CI 29.4–49.6), and diarrhoea (23.7%, 95% CI 15.6–33.4) as shown in Table 1. The mean haemoglobin was 11.9 g/dl (±3.0), whereas the overall HIV status was 19.3% (95% CI 10.5–26.6). There were no significant differences between males and females in terms of presenting symptoms, HIV status and mean haemoglobin levels.

**Table 1:** Demographic characteristics and presenting symptoms of study participants, stratified by gender

	Overall	Female number (%)	Male number (%)	p value^a^
Gender [n (%)]	97	45 (46.4)	52 (53.6)	0.54
Age in years [Mean (SD)]	46.6 (15.9)	49.1 (16.7)	44.4 (14.9)	0.15
Area of residence [n (%)]				0.22
Low cost	49 (50.5)	22 (48.9)	27 (51.9)	
Medium cost	27 (27.8)	10 (22.2)	17 (32.7)	
High cost	21 (21.7)	13 (28.9)	8 (15.4)	
Occupation status				0.01
Unemployed	52 (53.6)	31 (68.9)	21 (40.4)	
Employed	45 (46.4)	14 (31.1)	31 (59.6)	
Presenting symptoms				
Abdominal pain				0.88
Yes	59 (60.8)	27 (60.0)	32 (61.5)	
No	23 (39.2)	18 (40.0)	20 (38.5)	
Blood in stool				0.50
Yes	38 (39.2)	16 (35.6)	22 (42.3)	
No	59 (60.8)	29 (64.4)	30 (57.7)	
Diarrhoea				0.80
Yes	23 (23.7)	10 (22.2)	13 (25.0)	
No	74 (76.3)	35 (77.8)	39 (75.0)	
Vomiting				0.39
Yes	16 (16.5)	9 (20.0)	7 (13.5)	
No	81 (83.5)	36 (80.0)	45 (86.5)	
Weight loss				0.08
Yes	18 (18.6)	5 (11.1)	13 (25.0)	
No	79 (81.4)	40 (88.9)	39 (75.0)	
Fever				0.72
Yes	8 (8.2)	3 (6.7)	5 (9.6)	
No	87 (91.8)	42 (93.3)	47 (90.4)	
HIV				0.95
Yes	17 (19.3)	8 (19.1)	9 (19.6)	
No	71 (80.7)	34 (80.9)	37 (80.4)	
Anaemia				0.98
Yes	44 (51.2)	20 (51.3)	24 (51.1)	
No	42 (48.8)	19 (48.7)	23 (48.9)	

^a^Except for the age, where a *t* test was used, the p value was derived with the Chi square test, including Fisher’s exact test where appropriate

### Carriage rates and diagnostic yield

Mycobacteria species were isolated in 13 participants (13.4%, 95% CI 6.5–20.3) out of which NTM were isolated in 7 (7.2%, 95% CI 1.9–12.4) of the participants. *M. tuberculosis* was isolated in 6.2% (95% CI 2.3–13.0) of the participants. There were no differences in carriage rates between males and females. Descending colon samples were the most likely to be positive (9.8%, 95% CI 3.7, 15.8) followed by stool samples (6.8%, 95% CI 1.0–12.6), caecal biopsy (6.1%, 95% CI 0.3–11.8) and intestinal lavage samples (5.9%, 95% CI 0.3–11.5) as shown in Table 2. The contamination rate was 17.6%.

**Table 2:** Diagnostic yield for *Mycobacterial* spp. from abdominal specimens among endoscopy patients attending endoscopy clinic at University Teaching Hospital in Zambia

Specimen	Total number isolated (%)	Species isolated	Diagnostic yield (%)
Descending colon biopsy	9	4 MTB 2 *Mycobacterium gordonae* *2 Mycobacterium kansasii* *1 Mycobacterium genavense*	9.8 (95% CI 3.7–15.8)
Stool	5	3 MTB 2 *Mycobacterium gordonae*	6.8 (95% CI 1.0–12.6)
Caecal biopsy	4	1 MTB 1 *Mycobacterium kansasii* *2 Mycobacterium gordonae*	6.1 (95% CI 0.3–11.8)
Intestinal lavage	3	2 MTB 1 *Mycobacterium gordonae*	5.9 (95% CI 0.3–11.5)

Sample size for the participants was n = 97

Sampling was random among consenting respondents

No participant carried more than one species of mycobacteria at a time. However, in 3/69 participants (4.3%) we isolated mycobacteria in both stool and descending colon biopsy. Three other patients (4.3%) also produced mycobacterium isolates from both caecal biopsies and descending biopsy specimens.

The intestinal carriage of mycobacteria was not associated with age, sex or presenting symptoms such as diarrhoea, abdominal pain, weight loss, rectal bleeding, anaemia as well as HIV status. This is shown in Table 3.

**Table 3:** Relationship between carriage of Nontuberculous mycobacteria and *M. tuberculosis* and characteristics of endoscopy patients at the University Teaching Hospital, Lusaka

Variable	Nontuberculous mycobacteria					Mycobacterium tuberculosis				
	Number N = 97	Crude OR		Adjusted OR		Number N = 97	Crude OR		Adjusted OR	
		OR (95% CI)	p value	OR	p value		OR (95% CI)	p value	OR	p value
Sex										
Female	4	1		1		2	1		1	
Male	3	0.63 (0.13–3.00)	0.55	0.28 (0.04–1.94)	0.20	4	1.79 (0.31–10.40)	0.98	0.34 (0.03–3.89)	0.38
Age										
Below 45 years	6	1		1		5	1		1	
Above 45 years	1	0.16 (0.02–1.45)	0.06	0.22 (0.02–2.08)	0.26	1	5.11 (0.55–47.4)	0.11	–	^–^
Occupational status										
Unemployed	2	1		1		2	1		1	
Employed	5	3.12 (0.56–17.41)	0.17	3.64 (0.53–24.99)	0.37	4	2.44 (0.42–14.26)	0.31	7.05 (0.31–162.40)	0.22
Weight loss										
No	6	1		1		5	1		1	
Yes	1	0.72 (0.08–6.42)	0.76	1.67 (0.14–20.38)	0.69	1	0.87 (0.09–8.03)	0.90	–	
Anaemia										
No	1	1		1		5	1		1	
Yes	6	6.47 (0.70–59.77)	0.06	5.75 (0.54–61.01)	0.15	1	1.17 (0.01–1.62)	0.08	0.08 (0.01–1.20)	0.07
HIV										
No	6	1		1		3	1		1	
Yes	1	0.68 (0.07–6.11)	0.73	1.35 (0.12–14.96)	0.81	1	1.42 (0.14–14.74)	0.77	4.84 (0.22–04.87)	0.31

## Discussion

In this study, we found evidence of NTM among patients booked for colonoscopy and flexible sigmoidoscopy at UTH. This observed prevalence is higher than a previous report in the stools of patients with chronic diarrhoea [18] in the same hospital. The reasons for this are unclear and were beyond the scope of this study, but the development of MGIT detection systems in the interval between these studies may partly explain the higher apparent prevalence. However, much higher prevalences have been detected from other Zambian patient populations using sputum samples, where the prevalence of NTM was as high as 56% [13, 19]. Given the known biological characteristics of these micro-organisms, their presence in this population also suggests an environmental contamination.

This being a hospital based study, there are inherent external validity reservations for such findings, as this study was limited to a highly selected population scheduled for endoscopy. There may be arguments supporting the thinking that external validity can only be guaranteed if such a study should have been done in the general population in Lusaka. We argue that the great majority of our participants were generally healthy, apart from having abdominal symptoms for which they sought endoscopy services. It would not be possible to justify colonoscopy in unselected patients in the general population, merely for the purpose of obtaining biopsies. Notwithstanding these limitations, we think that these findings may even be an under-estimate of the burden of NTM. This is due to the use of the culture methods without special incubation temperatures or additional nutritional supplements to culture more fastidious NTM [20] from both the clinical and water samples. Further, the use of *N*-acetyl l-cysteine—6% NaOH (NALC–NAOH) for decontamination may have been overly harsh for some samples with low levels of organisms [21]. Our results were not affected by non-participation due to refusal to give consent as this remained below 5%. We consider it likely that this population has a measurable carriage rate of NTM in the intestine.

Finding that the carriage of NTM was not associated with the gender of the respondent, age, employment status, or presenting symptoms was not surprising given the ubiquitous nature of these organisms. These results illustrate the abundant nature of NTM, which are found in soil, dust and water systems [22]. This might explain the general exposure that exists irrespective of the age, sex and other patient characteristics. However, previous studies elsewhere have shown a relationship between isolation of NTM and both gender and age of the patient [6, 23] and type of occupation [24]. Tests for NTM done on the hospital water systems during the period of the study were found to be negative. Given that the water samples and the clinical samples were processed in the same way, our findings suggest that the NTM were not due to contamination.

It has been suggested that NTM, such as the *Mycobacterium avium* complex, may be responsible for some non-specific abdominal symptoms such as anorexia, weight loss, dysphagia, odynophagia and abdominal pain [11]. While abdominal pain was the most common presenting symptom in our patients (72.0%), there was no relationship between the carriage of NTM and presenting symptoms such as abdominal pain, weight loss, vomiting, nausea, dizziness and anorexia. We did not isolate *Mycobacterium avium* species in our patients.

We found a higher diagnostic yield of NTM from colonic biopsies compared to stool and lavage samples, although this was not statistically significant. *Mycobacterium avium* is known to be the most frequently isolated NTM from the gastrointestinal tract among immunocompromised patients [25]. However, this was not the case in this study because *Mycobacterium gordonae* was the most frequently isolated species in our patients, followed by *M. Kansasii* and *M. genavense.* All the isolated species were potentially pathogenic [26]. Reports of increasing incidence of nontuberculous mycobacteria point to the need for further attention to these organisms [3, 27].

It is well known that immune-compromised patients are more likely to suffer from disease caused by NTM than the general population. Carriage rates of NTM were not associated with levels of haemoglobin and HIV status in our patients. The HIV prevalence was 19.3%, and the patients were generally healthy with very few who were chronically ill or had other debilitating illnesses. Compared to the general Zambian adult population, whose HIV prevalence according to the Zambia Demographic and Health Survey (ZDHS) [28] was 13.3% in 2014, the HIV prevalence in our population was higher.

Some of our patients had *M. tuberculosis* isolates. However, only one patient (who was not suspected to have TB at the time) showed endoscopic findings that were consistent with abdominal tuberculosis in the ileo-caecal area. Culture of the caecal biopsy was positive for *M. tuberculosis* and he was subsequently treated for abdominal TB. Other patients were referred to the appropriate TB services for further evaluation.

## Conclusion

Our results have demonstrated presence of NTM in this population, suggesting an environmental contamination of the gut by potentially pathogenic nontuberculous mycobacteria that was not associated with any symptoms or demographic status. The extent to which these organisms are responsible for some of the morbidity in our patients remains to be unravelled. Given that this may be an indicator or a bigger burden of NTM in this population, there is thus need to further explore this burden and the contribution it could have on the disease in the intestine as well as critically examining potential factors that might be important predictors. We found that descending colon biopsies gave the highest yield for detection of NTM.

## Abbreviations

HIV: human immunodeficiency virus
MGIT: mycobacteria growth indicator tube
NTM: nontuberculous mycobacteria
UTH: University Teaching Hospital
